# Medical Jurisprudence

**Published:** 1826-01

**Authors:** 


### Medical Jurisprudence.

1. *Royal College of Surgeons.** In the first number of this series, pp. 241—2, we took the liberty of making some comments on the bye-law of the College of Surgeons, which limited the tuition of anatomy and surgery to certain classes, by refusing the certificates of other teachers than those within the prescribed channel. After offering every palliative reason that we could think of for such an apparently monopolizing measure, we hesitated not to aver that the said bye-law was pregnant with mischief, if enforced; as it would "shut the door on merit, unless that merit were conjoined with fortune and friends." "No principle of decay (we then observed) is more certain or fatal, than that which results from *security* of monopoly. To withdraw competition from any pursuit or undertaking, moral or physical, is to sap the foundation of human energy, and foster indolence." We were told, at that time, by one of the most distinguished Members of the College, that the bye-law was merely a weapon to *prevent* the introduction of *incompetent teachers* into private schools of anatomy and surgery, and that it was never meant to be put in operation, excepting under such circumstances. With this explanation we were satisfied, and said no more on the matter; but it appears that our words were prophetic—and that this obnoxious law has actually been called into force to prevent two gentlemen (who appear to be well qualified) from commencing as lecturers on anatomy and surgery in this metropolis!—Connected, as we are, by ties of friendship and professional intercourse, with some distinguished Members of the College, it must be very unpleasant to animadvert on any of the laws which emanate from such a body. But public duty and moral principle compel us to notice and to comment on the medico-legal events of the day; and a year's reflexion does not induce us to alter our sentiments on the abstract question of the law to which we have just alluded. Of the private motives or merits of the parties on either side, we profess to know nothing—and that we can have a particle of self-interest in view, we defy our most inveterate adversaries to even insinuate. We shall, therefore, in the first place, put our readers in possession of the leading features of Dr. Armstrong's letter, and then add a few comments of our own.

It appears, from our author, that Mr. Bennett was educated in the University of Dublin, where he took the degree of Bachelor of Arts, in 1817. Having devoted six years to the study of medicine in all its branches, and become a Member of the Irish College, he subsequently spent three years in Paris, and there began, first to assist, and afterwards to teach, the English students resident in that metropolis. Having become popular there, he applied to the London College of Surgeons to have certificates of attendance on his lectures recognized by the Court

* Dr. Armstrong's Letter to the Members, &c. October, 1825.

of Examiners. This they refused. About this time, also, Mr. Bennett began to experience illiberality and annoyance from the French students, in consequence of which, he addressed a memorial to our Ambassador at Paris, begging his interference with the French Government. The Ambassador declined, and a memorial was next presented to Mr. Canning. Our Foreign Secretary referred Mr. B's. letter to the College of Surgeons, where, as might be readily expected, cold water was thrown on Mr. Bennett's petition, and Mr. Canning declined interfering. Thus situated, Mr. Bennett had no other resource than to return to his own country, with the view of commencing as a private teacher; but here new difficulties arose; for, although he shewed that he was a teacher prior to the promulgation of the bye-law, his certificates were not only refused, but it is said, in the letter before us, that he was personally insulted at the College.

These are the prominent facts of the case, as stated by Dr. Armstrong. We have not space to go into the animadversions, and somewhat severe strictures, which our author passes on the Court of Examiners of the College—contrasting their examinations with those which obtain in Paris and Dublin, and accusing them, in pretty round terms, of acting as a corporate body, entirely for their own interests, and with little regard to the good of science or of society at large.

Upon the general policy of the College, we have nothing to say; but in respect to the bye-law, which is the cause of all these altercations, we still entertain the same sentiments which we did at first—namely, that it is calculated to crush merit—promote family compacts—render the offices of teachers hereditary, without any regard to qualifications—foster indolence—and, finally, to do all in its power to overthrow surgical science itself.

At the same time, we are free to confess, that we do not blame the College for refusing to admit the certificates of a Parisian lecturer. We are not quite such cosmopolites as to consider the interests of foreign countries as perfectly synonymous with our own. Instead of encouraging the emigration to Paris, we should prefer endeavouring to remove the difficulties which lie in the way of anatomy in London. A day must come when our intercourse with the Continent will be interrupted by war, and then we must again fall back on our own resources, dilapidated, perhaps, by the current which sets in peace towards other countries. We think it would be proper, in the College of Surgeons, as is the practice with the College of Physicians, and the different universities, to admit a portion of time passed in studies out of the kingdom; but certainly they ought not to accept this portion, without the complementary portion being spent in their own country.

While we are on the subject of anatomy, we shall take the liberty of offering an opinion, founded on personal observation, that the dissecting rooms in Paris, do not turn out such good anatomists, as the abundance and cheapness of subjects might lead one to expect. This very abundance, coupled with cheapness, begets a careless and slovenly mode of dissecting, which must have struck every attentive observer, on entering the Parisian rooms. It is with dead bodies as with every thing else in this world. The scarcer and dearer the article, the more we prize it, and the more we endeavour to turn it to the best account. More real and useful anatomical knowledge may be acquired from the careful dissection of one subject, than from the rapid hacking and slashing of twenty carcases.

It is said by the able author of the pamphlet before us, that the dearth of subjects for dissection, in this country, has produced a paucity of operating surgeons. We are rather sceptical on this point. The anatomy which is necessary for the operating surgeon, is of the easiest kind, and may be acquired in two or three years by any one who is not a dunce. The relative situation of parts—the distribution of vessels and nerves—the origin, course, and insertion of muscles—the mechanical structure of articulations—and osteology, are the portions of anatomy which are essentially necessary for the operating surgeon. But how easy is it to acquire this knowledge, compared with that of *structure* itself! How many years does it require to become intimately acquainted with the structure of bone, muscle, cartilage, ligament, nerve, brain, viscera, glands, and the various tissues of the human body! These are the real difficulties of anatomy; but, fortunately, a man may be an excellent operator and a good physician, without this intimate knowledge of *ultimate* structure. The first, or easy part of anatomy is the essential part. The other *ought* to be acquired, but it is comparatively of little use at the bedside of sickness, or in the operating room. We believe, then, that a good operator springs from the first kind of anatomy, and *practice on the living body*. This practice can rarely be obtained but in hospitals or public institutions—hence the number of operating surgeons will depend more on the number of public institutions than on the facilities of anatomy. Is it not a fact, that all the great operations are now every day performed in the provincial towns, even where the greatest difficulty of procuring dead bodies prevails? To what is this to be attributed but to the increase of public institutions, and to the attention which is paid to dissection in the few bodies we possess? If these observations be correct, there is less danger to be apprehended from the paucity of subjects, than might at first sight appear. There are few evils in this world, which do not tend to their own correction, and this, we believe, is one of them, While, therefore, we deprecate the law which would stifle *competition*, we would recommend increased cultivation of the means we possess, rather than encourage emigration to other countries, where there are many circumstances that counterbalance the facilities of dissection. Among these we may reckon the illiberal and repulsive conduct of many of the French students. Among the crowd of paupers who rush to Paris for gratuitous education, it is almost impossible for an English gentleman to appear, without continual insult. This is a shame to the boasted politeness of the French nation! The English student, in London, who would debase himself by offering the slightest insult, or even disrespect, to a foreigner pursuing his studies here, would be hissed out of the class-room.

